# The impact of slip perturbations on minimum toe clearance during walking in younger and older adults

**DOI:** 10.1371/journal.pone.0323307

**Published:** 2025-05-23

**Authors:** Aaron M. Gelinne, Andrew D. Shelton, Jason R. Franz

**Affiliations:** 1 Department of Neurosurgery, University of North Carolina at Chapel Hill, Chapel Hill, North Carolina, United States of America; 2 Lampe Joint Department of Biomedical Engineering, University of North Carolina at Chapel Hill and North Carolina State University, Chapel Hill, North Carolina, United States of America; Aichi Prefectural Mikawa Aoitori Medical and Rehabilitation Center for Developmental Disabilities, JAPAN

## Abstract

**Purpose:**

The purpose of this study was to examine the kinematic differences in minimum toe clearance (MTC), a risk factor for falls, between older and younger adults during habitual walking and treadmill-induced slip perturbations.

**Materials and methods:**

Data from 28 older adults and 30 younger adults were analyzed for this study. Each subject was fitted with whole-body retroreflective markers for 3D motion capture and walked habitually and while responding to treadmill-induced slip perturbations. Minimum toe clearance and leg joint kinematics were obtained and compared between groups and across conditions.

**Results:**

There was no difference in MTC between age groups during habitual walking (Older: 5.75 ± 1.08 cm, Younger: 5.45 ± 0.93 cm, p = 0.125). Independent of age, MTC increased significantly in response to treadmill-induced slips (p < 0.001). However, significant age × condition interactions revealed that older adults increased MTC significantly less than younger adults in response to treadmill-induced slips. Older adults walked with significantly less knee flexion (31.4 ± 9.9° vs. 41.3 ± 11.9°; p < 0.001) and significantly more hip internal rotation (6.5 ± 6.3° vs. 3.8 ± 6.3°; p = 0.004) than younger adults during slip perturbations.

**Conclusions:**

This study builds on prior work to conclude that, compared to younger adults, older adults appear to have a diminished capacity to instinctively increase MTC during walking in response to slip-like balance perturbations, which may allow them lesser time to accommodate instability.

## Introduction

Fall-related trauma is a significant contributor to morbidity in older adults (age ≥ 65 years), leading to an enormous and increasing burden on the US healthcare system with our aging population[1,2]. The U.S Centers for Disease Control estimates that approximately 29% of older adults will suffer a fall, accounting for approximately 50% of all emergency department injuries and deaths in this population [3]. Additionally, people who suffer from falls are much more likely to have a fall in the future highlighting the need for further characterizing and identifying those at risk [4]. The etiology of falls is multi-factorial and arises from a combination of neurologic/balance disorders, cognitive impairment, muscle weakness, syncope, iatrogenic-induced illness, and/or visual impairment [5,6]. Despite their complex origins, most falls occur during locomotor activities such as walking for which alterations in gait parameters and control strategies may point to specific impairments and targets for intervention.

Given its relevance to safe mobility and protection against instability and falls, age-related differences in minimum toe clearance (MTC) have received significant attention in prior studies [7–9]. MTC is defined as the minimum vertical distance of the toe from the ground during leg swing in walking [10]. There are at least two potential consequences of lesser MTC during walking. First, lesser MTC can cause the foot to make contact with the ground or obstacle, precipitating instability or a trip [11]. Second, lesser MTC implies less time may be available to make swing limb adjustments or corrections in the presence of a balance disturbance prior to requisite foot contact. MTC has been shown to have increased variability due to gait pathology, such as diabetic neuropathy and peroneal neuropathy [12,13]. Conversely, although the evidence is mixed, several studies report that older adults without overt disease walk with altered joint kinematics that increase MTC compared to younger adults [14,15]. This may be interpreted as a more cautious strategy adopted by older adults to prevent falls.

While informative, a major component of falls in the community occurs outside of habitual locomotion and includes moments of gait challenges [16]. Balance perturbations are a class of experimental paradigms used based on growing evidence that even the most sophisticated balance outcome measures during habitual walking are unlikely to predict the way in which individuals recover from the instability that would precipitate a fall [17]. As a principal example, slip perturbations have been used to emulate gait challenges that may increase susceptibility to falls and a dynamic task in which MTC may be highly relevant for balance recovery in older and younger adults [18]. Indeed, some studies on patients with multiple sclerosis have shown that MTC decreases during a slip perturbation which may suggest a reduced time available to make neuromuscular changes necessary to accommodate slip-induced instability [19]. In this context, decreased margins for corrective motor adjustments to accommodate slip-induced instability and ultimately decrease the probability of falling is of critical importance to our understanding various gait changes with ageing and clinical pathology. However, no study to our knowledge has quantified age-related differences in MTC during tasks spanning habitual walking to slip perturbations. This gap is important to address; better understanding age-related differences in MTC, particularly those scenarios and circumstances in which these differences are disproportionately relevant, could point to potentially modifiable factors to inform fall prevention strategies or rehabilitation programs.

The purpose of the current study was to quantify lower extremity kinematics and minimal toe clearance in older and younger adults during habitual walking and while responding to slip perturbations. We first hypothesized that, compared to younger adults, older adults would habitually walk with increased minimal toe clearance. Second, we hypothesized that younger adults would afford themselves more time to accommodate slip-induced instability via increased MTC compared to older adults. Finally, we hypothesized that – independent of age – increased minimal toe clearance would be accompanied by increased hip, knee, and ankle joint flexion which would be further pronounced during slips.

## Methods

### Participants

This study was approved by the University of North Carolina Biomedical Sciences Institutional Review Board and all subjects provided written informed consent prior to participating. Participants were recruited using study flyers and online announcements. Inclusion/exclusion criteria were evaluated using telephone screening. Exclusion criteria, chosen to ensure subjects could complete the assigned walking trials, included recent lower extremity injury, known neurologic disorders that would impact ability to ambulate, current use of medications that would affect balance or mobility, those without normal or corrected to normal vision and use of assistive devices. Enrollment goals included relatively equal representation among female and male participants. A power analysis determined that n = 34 participants per group would have 80% power to detect (p < 0.05) an effect size of 0.7 for between-group differences in MTC during walking. Using a questionnaire to determine eligibility, we thus recruited 35 older adults (age: ≥ 65 years) and 34 younger adults (age: 18–35 years) to participate. After quality control for marker data integrity, 28 older adults (12 Females/16 Males) and 30 younger adults (15 Female/15 Male) were included in our analysis.

### Experimental protocol

All trials were performed in the subjects’ own shoes. We determined each subject’s preferred overground walking speed using the average of 4 timed 30-meter walks. We fit 48 retroreflective markers on participants’ pelvis, trunk, and left and right legs. Leg markers were placed on the sacrum, bilateral anterior superior iliac spines, posterior superior iliac spines, lateral femoral epicondyles, lateral malleoli, lateral calcanei, and lateral first and fifth metatarsal heads; torso were placed on the sternum, clavicle notch, seventh cervical vertebral prominence, and tenth thoracic vertebra. Rigid tracking marker clusters were affixed to subjects’ thighs and shanks.

Following marker placement, subjects walked on a force-sensing, dual-belt treadmill (Bertec Corp., Columbus, Ohio, USA) for two minutes at their preferred overground walking speed for acclimation. Subjects then completed one 2-min habitual walking trial and one trial that incorporated slip perturbations. For the latter, we employed a slip perturbation paradigm described in previous studies [20] to elicit walking instability and test the effects on MTC. Briefly, participants responded to a series of 200 ms, 6 m/s^2^ treadmill belt decelerations delivered at the instant of random heel strikes using a custom MATLAB script (MathWorks, Natick, MA). Following the 200 ms perturbation, the treadmill belt returned to the subject’s preferred overground walking speed over 200 ms at the same 6 m/s^2^. A total of four perturbations were delivered (i.e., two belt decelerations per leg) at random steps during the trial, which lasted less than 2 minutes total.

### Measurements, data analysis, and minimum toe clearance

**Fig 1:**
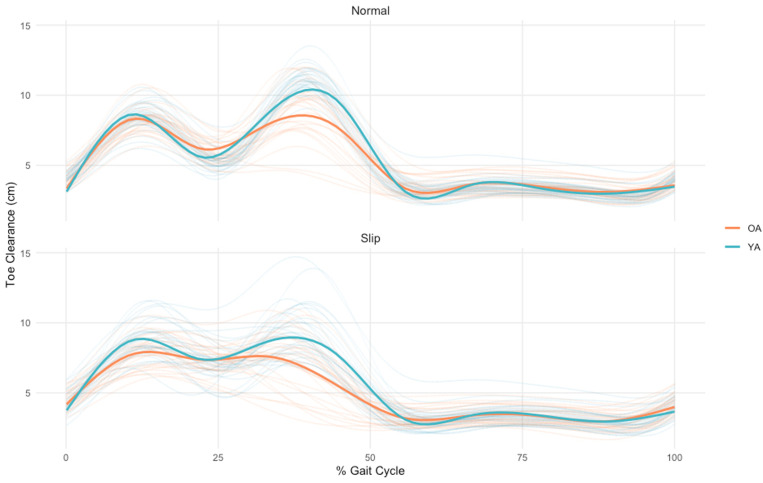
Group average (thick lines) and individual subject (thin lines) data showing first metatarsal vertical height for older (orange) and younger (blue) adults during habitual walking and in response to treadmill-induced slips over a gait cycle from toe-off to toe-off. Solid lines represent the average for each age group throughout the gait cycle. For the bottom panel, slip perturbations were delivered at the instant of heel-strike of the preceding step (not shown).

Marker data was collected at 100 Hz and filtered using a 4^th^ order Butterworth low-pass filter with a cut-off of 12 Hz. From these filtered marker trajectories, we calculated minimum toe clearance during the swing phase of both habitual walking and following slip perturbations. We defined a walking stride as the time between successive heel-strikes. We then determined minimum toe clearance from the first metatarsal marker using a custom MATLAB script. The toe trajectory during the swing phase of walking very consistently follows a bimodal curve (Fig 1). We determined minimum toe clearance as the local minima between the maximums associated with toe-off in early swing and heel strike in late swing. During habitual walking, this value was extracted for each swing leg per stride and then averaged bilaterally over the 2-minute trial to give an average MTC for each subject per habitual walking trial. We considered an average of all strides during habitual walking. For slip trials, MTC was taken for the subsequent stride immediately following heel strike after a perturbation was executed. All four perturbed strides were extracted and averaged bilaterally for analysis. Finally, we used a scaled musculoskeletal model in OpenSim (version 4.4, gait2392) and inverse kinematics to calculate time series of hip flexion and adduction, knee flexion, and ankle flexion. For the same strides analyzed for MTC, we extracted the values of these joint angles at the instant of MTC.

### Statistical analysis

We performed all statistical analysis in RStudio (Posit Software, Boston, MA). The primary outcome variable in this study was MTC. Explanatory variables included in the analysis were hip, knee, and ankle joint angles; age (older, younger) and condition (habitual/slip response). Continuous variables were confirmed for normality using a Shapiro-Wilk test and therefore parametric testing was utilized. All continuous variables are represented as mean ± standard deviation. Bivariate analysis was performed using pairwise t-test between explanatory and outcome variables. For primary comparisons, we used a mixed ANOVA with age as a between-subject effect and condition as a within-subject factor followed by Tukey’s post-hoc testing for pairwise comparisons. We repeated this analysis using an ANCOVA while controlling for preferred walking speed. Significance was determined using a p-value less than 0.05.

## Results

### Cohort analysis

Table 1 shows demographics for the subjects in this study. The mean age of younger adults was 22.4 ± 3.1 years and in older adults it was 73.0 ± 5.9 years. We found no significant differences in body mass or height between older and younger adults. However, older adults (1.19 ± 0.19 m/s) preferred a significantly slower walking speed than younger adults (1.34 ± 0.12 m/s; p < 0.001).

**Table 1:** Subject demographics†.

	Younger Adults	Older Adults	p-value‡
Age (years)¶	22.4 (3.1)	73.0 (5.9)	–
Height (m)	1.73 (0.08)	1.70 (0.11)	0.334
Body Mass (kg)	67.2 (19.6)	71.6 (19.6)	0.284
PWS (m/s)	1.34 (0.12)	1.19 (0.19)	**<0.001**

† Continuous variables represented as mean with standard deviation in parenthesis.

‡ Statistical analysis performed in R Studio using unpaired t-test for continuous variables. Significance determined at the p < 0.05 level.

¶ Variable controlled for as part of study design.

PWS: preferred walking speed.

### Minimum toe clearance

**Fig 2:**
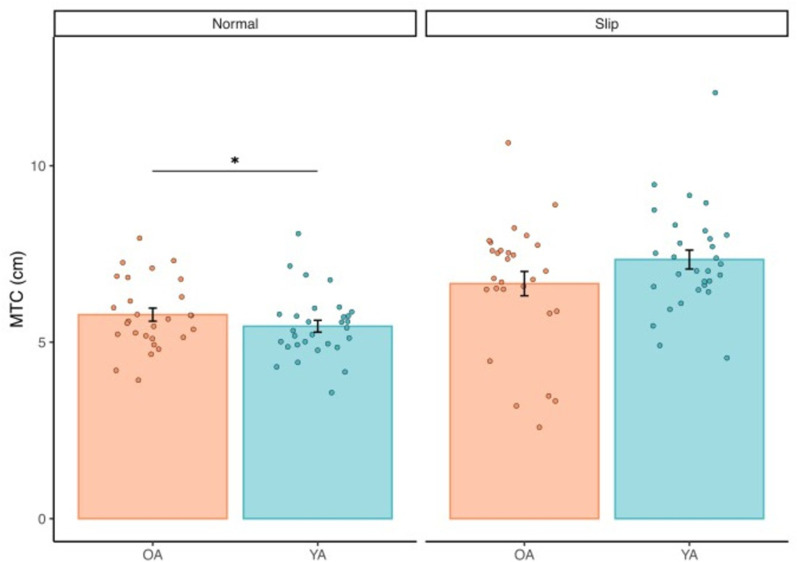
Average (standard error) minimal toe clearance (MTC) for older (OA, orange) and younger (YA, blue) adults during habitual walking and in response to treadmill-induced slip perturbations. Individual subject data are represented by filled circles. Asterisks (*) indicate significant differences between younger and older adults.

Figs 1 and 2 and Table 2 summarizes MTC outcomes for both walking conditions in younger and older adults. We found a significant main effect of condition on MTC (Table 3; p < 0.001) which, compared to habitual walking, averaged 15% (p < 0.001) and 34% (p < 0.001) larger when responding to treadmill-induced slips for older and younger adults, respectively. We did not find a significant main effect of age on MTC (p = 0.571). However, a significant age × condition interaction effect revealed that younger adults increased their MTC in response to treadmill-induced slips more than twice that in older adults. This was supported by an independent-samples comparison showing that ΔMTC (perturbation-habitual) was significantly larger in younger versus older adults (p = 0.002). The main effect of condition (p = 0.836) was no longer significant when controlling for preferred walking speed. However, the significant age × condition (p = 0.024) interaction persisted and continued to reveal that older adults increased MTC significantly less than younger adults in response to treadmill-induced slips.

**Table 2:** MTC and joint angles during leg swing grouped by test condition and age†.

	Habitual			Slips		
	Younger	Older	p-value	Younger	Older	p-value^‡^
MTC (cm)	5.45 (0.93)	5.75 (0.97)	0.125	7.28 (1.45)	6.62 (1.78)	0.104
ΔMTC (cm)	--	--	--	1.84 (0.97)	0.87 (1.3)	**0.002**
Joint (degrees)						
Hip Internal Rotation	4.6 (6.2)	6.1 (6.1)	0.123	3.8 (6.3)	6.5 (6.3)	**0.004**
Hip Adduction	11.0 (7.1)	8.3 (5.0)	**0.015**	8.1 (6.7)	6.1 (5.0)	0.074
Hip Flexion	44.9 (11.1)	43.2 (9.2)	0.395	47.1 (10.6)	44.5 (9.5)	0.174
Knee Flexion	51.5 (7.9)	49.5 (8.2)	0.186	41.3 (11.9)	31.4 (9.9)	**<0.001**
Ankle Plantarflexion	4.9 (4.3)	4.9 (3.9)	0.990	6.2 (4.7)	5.2 (4.2)	0.286

† Continuous variables represented as mean with standard deviation in parenthesis.

‡ Statistical analysis performed in R Studio using unpaired t-test for continuous variables. p-values represent difference between younger and older adults for each test condition. Significance determined at the p < 0.05 level.

MTC: minimum toe clearance.

**Table 3:** Two-way ANOVA for interaction between age and test condition on MTC.

Variable	Sum Squares	Df	Mean Square	F value	p-value^‡^
Age	0.0001	1	0.46	0.325	0.571
Condition	0.005	1	0.005	80.97	**<0.001**
Interaction	0.001	1	0.001	10.35	**0.002**
Residuals	0.004	56	0.00007		

‡ Statistical analysis performed in R Studio using a mixed ANOVA test with test condition as a within subject and age as between subject effects. P-values represent difference between effects. Significance determined at the p < 0.05 level.

MTC: minimum toe clearance.

### Joint kinematics

**Fig 3:**
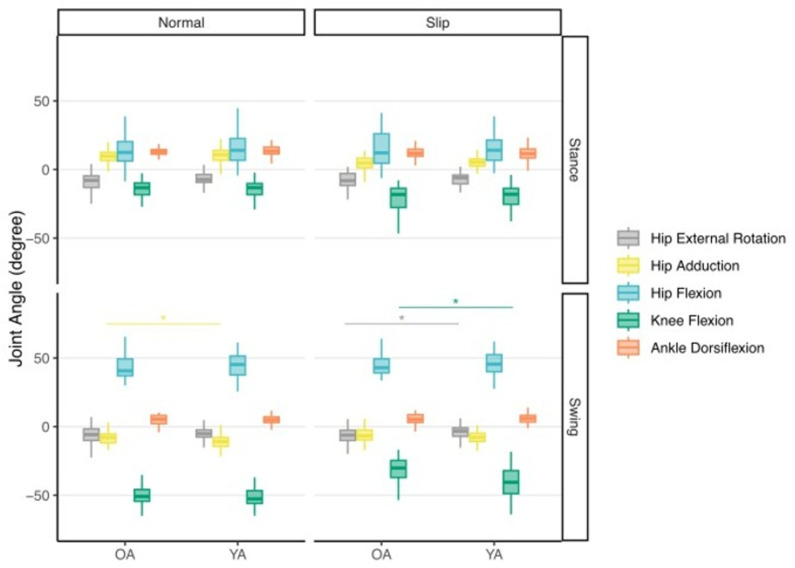
Box plot showing median hip, knee, and ankle joint angles at the instant of minimum toe clearance during habitual walking and in response to treadmill-induced slip perturbations for the swing leg (ipsilateral) and stance leg (contralateral). Asterisks (*) indicate significant differences (p < 0.05) between younger and older adults for the color associated with respective joint angles. OA: older adults; YA: younger adults.

Fig 3 and Table 2 summarize joint kinematic outcomes at the instant of MTC for both walking conditions in younger and older adults. Age-related differences in joint angles differed between habitual walking and when responding to slip perturbations. During habitual walking, older adults only walked with lesser hip adduction at the instant of MTC than younger adults (8.3 ± 5.0 vs. 11.0 ± 7.1°; p < 0.001). Conversely, in response to treadmill-induced slips, older adults walked with significantly less knee flexion (31.4 ± 9.9° vs. 41.3 ± 11.9°; p < 0.001), and significantly more hip internal rotation (6.5 ± 6.3° vs. 3.8 ± 6.3°; p = 0.004) than younger adults. However, we found no significant between-condition differences in joint angles for older or younger adults.

## Discussion

Researching age-related differences in MTC during various locomotor activities is important; lesser MTC: (i) can cause the foot to make contact with the ground or obstacle, and (ii) implies less time may be available to make swing limb adjustments or corrections in the presence of a balance disturbance prior to requisite foot contact. The primary findings of this study are three-fold: 1) In contrast to our first hypothesis, MTC was not significantly different between older and younger adults during habitual walking, 2) In support of our second hypothesis, MTC increased substantially more in younger than in older adults in response to a slip perturbation, and finally 3) In contrast to our third hypothesis, higher MTC during a slip perturbation was not accompanied by between-condition differences in joint kinematics and thus likely arises through a combination of smaller deviations spanning multiple joints. These findings, as we outline in more detail below, may have implications for the development of techniques used to identify populations at risk of falling and/or to introduce potentially modifiable factors for gait training to prevent falls.

### Minimum toe clearance in older and younger adults during habitual and perturbed walking

In contrast to some [14,15] but not all [21] prior reports, we found that MTC was not larger in older adults compared to younger adults during habitual walking. Moreover, both younger and older adults significantly and instinctively increased MTC in response to a slip perturbation (Fig 1). However, this instinctive increase was more than two-times greater in younger adults. The etiology of prior reports for higher MTC in older adults have been attributed to multiple characteristics of gait in older adults. For example, some studies have suggested that older adults walk with increased MTC as an adapted guard mechanism to prevent tripping/falling [14,22]. Others have shown that, like many other outcomes during walking, older adults who fall have greater step-to-step variability in MTC. Walking with increased MTC could mitigate the consequences of this variability, which would decrease the probability of inadvertent foot contact in a given swing phase [14]. The lack of a significant between-group differences in MTC for our cohort may reflect their relatively high level of fitness and physical activity levels. Though, as we discuss in more detail later, this further underscores the importance of our primary discovery that older adults appear to have a reduced capability to augment minimal toe clearance in response to a slip perturbation.

Although some previous studies have found a strong association between ankle dorsiflexion and MTC [23,24], ankle dorsiflexion was not a significant contributor to MTC in our study. This held true also when responding to a slip perturbation, during which older adults exhibited increased hip internal abduction and reduced knee flexion compared to younger adults (Fig 3). This is not necessarily a novel finding. For instance, Levinger et al. (2012) found that individuals with knee osteoarthritis regulated MTC via changes in knee flexion and hip abduction – similar to the between-group kinematic determinants we report here [25]. This suggests that, compared to older adults, the muscles contributing to knee flexion appear to be called upon in younger adults to increase MTC in the presence of a balance disturbance during walking. This may allow for an increased margin for corrective motor adjustments to accommodate slip-induced instability and ultimately decrease the probability of falling. That older adults may not be able to benefit from these increased margins should be considered in future work investigating mechanisms of instability and falls. Furthermore, the mean vertical toe trajectories shown in Fig 2 do suggest that despite similar MTC during slips between older and younger adults, there are other phases of leg swing in which older adults have a much lower toe clearance. We suspect that although some older adults may walk with a more guarded gait habitually, this deteriorates in the presence of a balance challenge requiring rapid reactive control. This is consistent with what is understood about age-related declines in neurologic function in response to stimuli [26,27].

### Clinical and translational implications

From a clinical perspective, the use of laboratory-based perturbations can be used to identify biomarkers of instability that may help to identify those at risk of falls. Indeed, understanding who is at risk and which features of gait are most responsible for that risk is the first step to preventing falls and subsequent injury. However, our recent work in older adults suggests that the response to walking balance perturbations is context specific; the structure of instability elicited by one context of perturbation may differ in functionally relevant ways from that elicited by a different context [18]. Our results may have implications for intervention as well. Indeed, interventions for fall prevention may focus on enhancing the instinct of older adults to increase MTC in response to unexpected perturbations, thereby increasing the time they have to make neuromuscular corrections. Given the joint-level determinants of MTC during slips, muscles contributing to knee flexion in particular could be targeted for gait training to increase foot clearance and allow for improved margins for corrective motor adjustments. In addition, our findings also set the stage for studies of fall risk in those with more overt gait pathology. Several vascular and neurologic diseases have been shown to reduce MTC and alter lower extremity kinematics including spinal disorders, peripheral vascular disease, and peripheral neuropathies [13,28–30]. Individuals with these diagnoses are also at a disproportionate risk of falling [31,32]. However, most of the available data are observational in nature and there is a lack of mechanistic evidence from the use of walking balance perturbations in these patient populations. Further understanding MTC under the influence of balance perturbations could help understand why these patients fall as well as guide rehabilitation efforts for prevention.

### Limitations

It is important to recognize some limitations of the current study. First, the head of the first metatarsal was utilized as the toe marker to calculate MTC which varies slightly from the point of the shoe. This was chosen as part of our standard marker protocol and is felt to be proportional to actual toe trajectories. In addition, we studied relatively healthy and physically active older adults. Thus, the age-related differences reported here may not generalize to older adults with more overt walking ability limitations or risk of falls.

## Conclusion

This study builds on prior work to conclude that, compared to younger adults, older adults appear to have a diminished capacity to instinctively increase MTC during walking in response to slip-like balance perturbations, which may allow them lesser time to accommodate instability. These data may be useful for identifying populations at risk of falling and guiding gait training to prevent falls in these groups.

## Supporting information

S1 File SupportingData_MTC. (XLSX)
